# The patient had a normal magnetic resonance imaging and temporal lobe epilepsy secondary to a porencephalic cyst but showed structural lesions (hippocampal sclerosis)^☆^

**DOI:** 10.1016/j.ebcr.2013.08.005

**Published:** 2013-09-28

**Authors:** Teppei Matsubara, Satoshi Ayuzawa, Tsukasa Aoki, Ayataka Fujiomto, Satoru Osuka, Akira Matsumura

**Affiliations:** a Department of Neurosurgery, Tsukuba University Hospital, Japan; b Department of Neurosurgery, Graduate School of Comprehensive Human Sciences, University of Tsukuba, Japan; c Department of Neurosurgery, Ryugasaki Saiseikai Hospital, Japan; d Department of Neurosurgery, Seirei Hamamatsu General Hospital, Japan

**Keywords:** Electrocorticography, Hippocampal sclerosis, MRI-negative temporal lobe epilepsy, Porencephalic cyst, Temporal lobectomy

## Abstract

Patients with a porencephalic cyst frequently develop intractable temporal lobe epilepsy (TLE). We report a surgically-treated male patient with intractable mesial TLE (mTLE) secondary to a porencephalic cyst. Although magnetic resonance imaging showed no hippocampal abnormalities, long-term video-electrocorticography revealed seizure onset discharges in the hippocampus. Temporal lobectomy brought an end to the patient's seizures. Hippocampal sclerosis was histopathologically confirmed (dual pathology). Careful evaluation of hippocampal epileptogenicity is required, and temporal lobectomy, which is less invasive than hemispherectomy, can be a treatment of choice for patients with mTLE secondary to a porencephalic cyst.

## 1 Introduction

Patients with a porencephalic cyst frequently develop epilepsy. In these patients, the traditional neurosurgical approach has been hemispherectomy because of the difficulty in localizing precise epileptogenic foci [1]. Although hemispherectomy is highly successful at eliminating seizures, it also shows higher morbidity than temporal lobectomy [2], [3], [4], [5].

Recent studies have shown that hippocampal sclerosis (HS) frequently coexists with porencephalic cysts, and these patients with intractable temporal lobe epilepsy (TLE) are good surgical candidates for temporal lobectomy [1], [3], [6]. The coexistence of HS with extrahippocampal lesions with epilepsy has been described as dual pathology and requires detailed presurgical evaluation to localize the epileptogenic focus [7].

Determining the optimal treatment for patients who present with TLE and negative magnetic resonance imaging (nMRI) is more challenging. We successfully treated a patient with intractable nMRI TLE secondary to a porencephalic cyst with temporal lobectomy, after a useful presurgical electrocorticography (ECoG) to localize the precise epileptogenic focus, avoiding invasive hemispherectomy.

## 2 Case report

A 29-year-old right-handed man suffered from intractable epilepsy from the age of 12. Habitual seizures manifested as loss of consciousness, staring, and automatism, followed by postictal confusion. Seizures occurred several times a week and were not controlled by 6 prior antiepileptic drugs. The patient had a history of lumbar myelomeningocele, which was surgically treated soon after his birth. At 26 years of age, he underwent resection of a lumbar lipoma and filum terminale. He had no history of neonatal asphyxia, febrile convulsion, or head trauma. He had normal mental status and no hemiplegia.

**Fig. 1:**
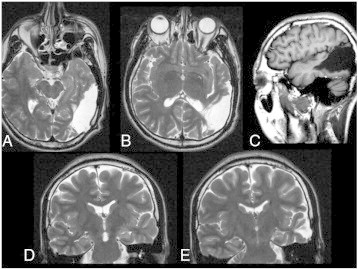
MRI shows a large porencephalic cyst in the left parietotemporal region with atrophic changes in the surrounding cortex (A, B; axial T2 weighted-imaging, C; sagittal T1-weighted imaging), but no atrophy or abnormal signal alteration is seen in the bilateral hippocampi (D, E; coronal T2-weighted imaging).

**Fig. 2:**
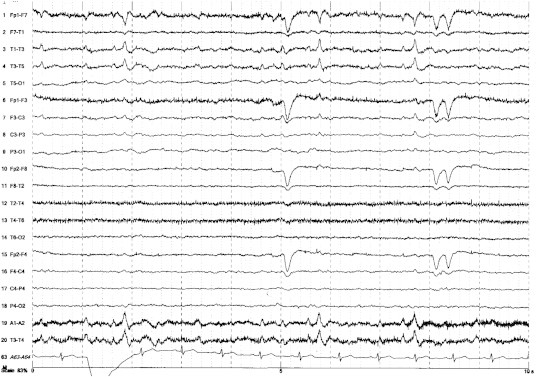
Long-term EEG shows interictal epileptiform discharges in the left anterior temporal region.

Magnetic resonance imaging revealed a large porencephalic cyst in the left parietotemporal region with atrophic changes in the surrounding cortex, no cortical dysgenesis, and, importantly, no atrophy or abnormal signal alteration in the bilateral hippocampi (Fig. 1). Long-term video-electroencephalography (EEG) monitoring revealed interictal epileptiform discharges in the left anterior temporal lesion (Fig. 2); however, no ictal onset could be localized. Interictal ^99m^Tc-ethyl cysteinate dimer (^99m^Tc-ECD) single-photon emission computed tomography (SPECT) revealed decreased cerebral blood flow in the mesial left temporal lobe. Ictal ECD SPECT was not performed because of technical limitations.

**Fig. 3:**
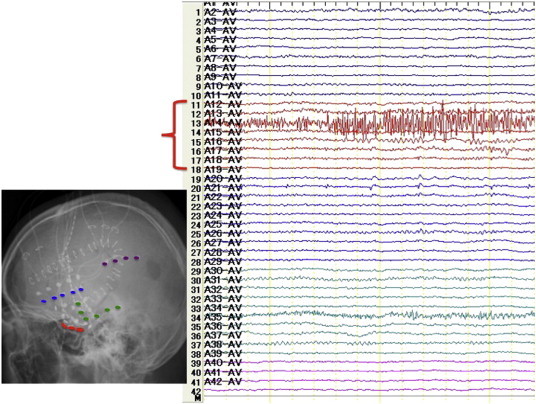
Long-term ECoG shows ictal discharges originating from the left mesial temporal region (red) and not in the area surrounding the porencephalic cyst (purple) nor the lateral mesial temporal region (green).

**Fig. 4:**
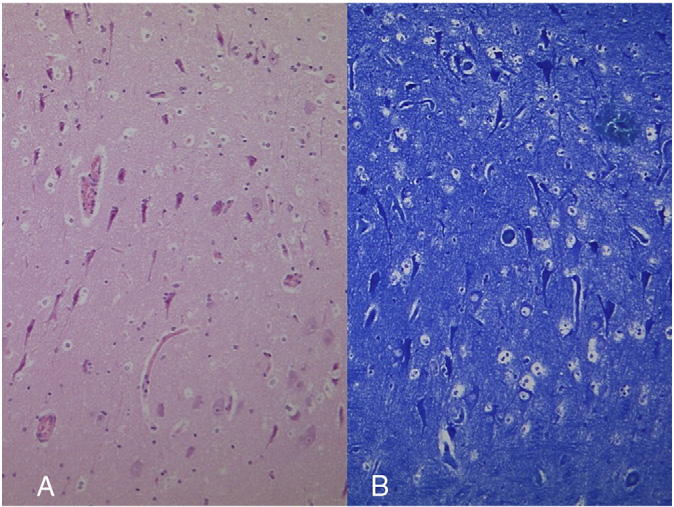
Histopathological examination reveals hippocampal neural loss at the CA1 area and granule cell dispersion, confirming a diagnosis of grade II–III HS. (A; HE × 100, B; Kleihauer–Betke (KB) stain × 100).

Although clinical features, EEG, and interictal SPECT suggested that the patient's seizures were due to mesial TLE (mTLE), MRI showed no abnormalities in the hippocampus, suggesting that the porencephalic cyst was the potential epileptogenic focus. To further determine the epileptogenic focus, long-term ECoG was performed, using subdural grid electrodes covering the left mesial temporal region, lateral temporal cortex, and parietal cortex surrounding the porencephalic cyst. We recorded 3 seizures over 7 days. All ictal discharges originated from the left mesial temporal region, and none originated in the area surrounding the porencephalic cyst (Fig. 3). We concluded that the left hippocampus was the epileptogenic focus. We performed left anterior temporal lobectomy and amygdalohippocampectomy. Histopathological examination revealed hippocampal neural loss at the CA1 area and granule cell dispersion, confirming a diagnosis of grade II–III HS (Fig. 4) [8]. Five years after surgery, the patient is completely seizure-free (Engel class I) without any morbidity.

## 3 Discussion

Porencephalic cysts occur as a component of vascular cerebral infarction during the prenatal or perinatal period and commonly manifest as congenital hemiplegia, intellectual impairment, and epilepsy [1]. It is generally assumed that the seizures originate in proximity to the porencephalic lesion, and surgical intervention is often discouraged because of the difficulty in accurately localizing the seizure foci [6]. Thus, hemispherectomy is a logical approach for seizure control [3].

However, if the seizure manifestation and scalp and further invasive ECoG suggest a specific epileptogenic focus in a patient with a porencephalic cyst, a more restricted resection might be preferable in view of anticipated motor function losses and perioperative morbidity and mortality [9].

The seizure semiology, interictal EEG, and interictal SPECT of the present patient suggested mTLE, but MRI findings showed no abnormalities in the hippocampus, i.e., hippocampal atrophy or signal alterations indicative of HS. Thus, we could not exclude the possibility that epileptogenic discharges around the porencephalic cyst propagated to the left mesial temporal lobe. Subsequent ECoG showed that the epileptogenic focus was the hippocampus and not the area surrounding the porencephalic cyst. Thus, we performed temporal lobectomy, which effectively eliminated the patient's seizures. Histopathology revealed mild HS.

To the best of our knowledge, there have been a total of 4 reports on 14 patients undergoing temporal lobectomy due to intractable TLE secondary to a porencephalic cyst (Table 1) [1], [3], [6], [10]. All 14 cases had a good clinical course, and HS was histopathologically confirmed. Of these 14 cases, ECoG confirmed an epileptogenic focus in the hippocampus in 4.

**Table 1:** Summary of reported patients with temporal lobe epilepsy secondary to porencephalic cyst undergoing temporal lobectomy.

Authors	Age/sex	Epileptogenic focus			Porencephalic lesion		Hippocampal features detected by MRI	Histopathological findings	Surgical outcome (follow-up months)
		Interictal EEG	Ictal EEG	ECoG	Side	Location			
Ho et al. (1997)	34/M	L temporal	Nonlocalizing	Not described^a^	Left	Frontoparietotemporal and basal ganglia	No visually detected HF abnormality	HS	Seizure-free (18)
	30/F	L temporal	Nonlocalizing	Not described^a^	Left	Temporoparietooccipital	L HF atrophy	HS	Seizure-free (18)
	15/M	Nonlocalizing	R temporal	Not described^a^	Right	Parietooccipital, inferotemporal, and thalamus	R HF atrophy and T2 hyperintensity on FLAIR	HS	Seizure-free (fewer than 6)
Ho et al. (1998)	44/F	Not described	Not described	Not described^a^	Left	Centroparietal	R HF atrophy and T2 hyperintensity on FLAIR	HS	Seizure-free (less than 6)
Burneo et al. (2003)	15/M	R temporal	R temporal	Not performed	Right	Hemispheric	R HS, and R brainstem atrophy	HS	Seizure-free (mean: 47, range: 22–67)
	29/F	L temporal	Muscle artifact	L subtemporal and lateral strips	Left	Parietal	L HS, and temporal atrophy	HS	Seizure-free (mean: 47, range: 22–67)
	31/M	L temporal	L temporal	Not performed	Left	Hemispheric	L HS, and temporal atrophy	HS	Seizure-free (mean: 47, range: 22–67)
	38/F	L temporal	L temporal	Not performed	Bilateral	Frontal	L HS, and temporal atrophy	HS	Seizure-free (mean: 47, range: 22–67)
	42/F	R temporal	R temporal	Not performed	Left	Centroparietal	R HS, and atrophic corpus callosum	HS	Seizure-free (mean: 47, range: 22–67)
Carreño et al. (2002)	Not described	Not described	Temporal	Not performed	Not described	Temporoparietal	HF atrophy	Not described	Auras only (72)
	Not described	Not described	Temporoparietooccipital	Not performed	Not described	Temporoparietooccipital	HF atrophy	Not described	Seizure-free (24)
	Not described	Not described	Temporal	Epidural electrodes	Not described	Frontotemporoparietal	HF atrophy	Not described	Auras only (96)
	Not described	Not described	Temporal and frontal	Epidural electrodes	Not described	Frontotemporoparietal	HF atrophy	Not described	Seizures persist (44)
Present case	29/M	L temporal	Not performed	L temporal	Left	Temporoparietal	No visually detected HF abnormality	HS	Seizure-free (60)

ECoG = electrocorticography; EEG = electroencephalography; F = female; HF = hippocampal formation; HS = hippocampal sclerosis; L = left; M = male; MRI = magnetic resonance imaging; R = right.

a One patient out of 4 underwent ECoG; however, the findings were not described.

Recent studies have emphasized dual pathology with HS and porencephalic cyst. Hippocampal sclerosis frequently coexists with a porencephalic cyst in patients with mTLE [1], [3], [6]. In addition, MR-based volumetry has revealed hippocampal atrophy in 95% of patients with porencephaly-related epilepsy [6], [11].

While most patients with mTLE may show the characteristic features of HS on MRI, including an atrophic hippocampus with a hyperintense signal on long repetition time sequences, up to 15% of patients with mTLE may have normal MRI [12]. This entity is the so-called nMRI TLE [13]. The treatment strategy for nMRI TLE remains controversial [14]. In the present patient, MRI was negative for HS, but histology confirmed mild HS. In a report by Immonen et al. [14], the surgical specimens of 26 (68%) of 38 patients with nMRI TLE undergoing temporal lobectomy did not display any pathological alteration, while 2 cases (5.2%) showed HS. Another report found that 38 (49%) of the 78 patients had mild to moderate HS, but no severe HS was identified [15]. The authors suggest that subtle pathology may not have been detected on MRI.

Of the 14 reported patients with porencephalic cyst and TLE (Table 1), 13 showed hippocampal atrophy on MRI, indicating HS. The remaining patient had no abnormal MRI findings in the hippocampus, but HS was confirmed by histopathological examination after temporal lobectomy, as in the present case.

We emphasize that the absence of radiological changes in the hippocampus does not preclude hippocampal epileptogenicity in mTLE with a porencephalic cyst. Thus, careful evaluation of hippocampal epileptogenicity is essential. Such cases can benefit from the less invasive temporal lobectomy rather than hemispherectomy.
